# Association of State-Level Firearm-Related Deaths With Firearm Laws in Neighboring States

**DOI:** 10.1001/jamanetworkopen.2022.40750

**Published:** 2022-11-08

**Authors:** Ye Liu, Michael Siegel, Bisakha Sen

**Affiliations:** 1 Department of Health Policy and Organization, School of Public Health, University of Alabama at Birmingham; 2 Department of Public Health and Community Medicine, Tufts University School of Medicine, Boston, Massachusetts

## Abstract

**Importance:**

Firearms are easily transported over state borders; hence permissive firearm laws in one state may have an interstate association with firearm-related deaths in nearby states.

**Objectives:**

To examine whether certain firearm laws have an interstate association with firearm-related deaths in nearby states.

**Design, Setting, and Participants:**

This cross-sectional observational study used data on state firearm-related deaths in the 48 contiguous states of the US between January 1, 2000, and December 31, 2019. A spatial autoregressive model with fixed effects for state and year was used to evaluate within-state, interstate, and overall associations between firearm laws and firearm-related deaths. Analyses were performed during January 2022.

**Exposures:**

The following 9 types of laws were evaluated: universal background checks for all firearms purchase, background checks for handgun sales at gun shows, license requirement to purchase all firearms, state dealer license requirement for handgun sales, requirement of retaining records of handgun sales, ban on purchasing a handgun on behalf of another, prohibition of firearm possession by persons who committed violent misdemeanors, required relinquishment of firearms for persons becoming prohibited from possessing them, and discretion in granting a concealed carry permit.

**Main Outcomes and Measures:**

State-level total firearm-related death rates, suicide rates, and homicide rates.

**Results:**

In sum, the study period included 662 883 firearm-related deaths of all intents. License requirement for firearm purchase had a within-state association (effect size, −1.79 [95% CI, −2.73 to −0.84]), interstate association (effect size, −10.60 [95% CI, −17.63 to −3.56]), and overall association (effect size, −12.38 [95% CI, −19.93 to −4.83]) per 100 000 population decrease in total firearm-related deaths. This law also had within-state association (effect size, −1.26 [95% CI, −1.72 to −0.80]), interstate association (effect size, −9.01 [95% CI, −15.00 to −3.02]), and overall association (effect size, −10.27 [95% CI, −16.53 to −4.01]) per 100 000 population decrease in firearm-related homicide.

**Conclusions and Relevance:**

The findings of this pooled cross-sectional analysis suggest that certain firearm laws in one state were associated with other states’ firearm-related deaths. Synergic legislative action in adjacent states, federal firearm legislation, and measures that reduce migration of firearms across state borders should be part of the overarching strategy to prevent firearm-related deaths.

## Key Points

**Question:**

How are states’ firearm laws associated with firearm-related deaths in nearby states?

**Findings:**

In this pooled cross-sectional analysis involving firearm laws and firearm-related deaths from 2000 to 2019 in the 48 contiguous states, a permit requirement for purchasing all firearms had an interstate association with decreased total firearm-related deaths and homicide, whereas the prohibition of firearm possession for individuals who have committed a violent misdemeanor had an interstate association with decreased firearm suicide.

**Meaning:**

These findings suggest that synergic legislative action to implement firearm laws in proximate states may help prevent firearm-related deaths.

## Teaser

This cross-sectional study analyzes the interstate association of firearm-related deaths with firearm laws in contiguous states.

## Introduction

Gun violence continues to be a major public health problem in the US. The years 2020 and 2021 have seen extraordinarily high numbers of gun-related fatalities.^1^ The response from the political leadership continues to be polarized, with Democrat lawmakers calling for more stringent gun regulations and Republican lawmakers dismissing the need for and the effectiveness of such regulations. On June 23, 2021, the Biden-Harris administration announced its strategy to combat gun violence using funding from the Rescue Act,^2^ a critical component of which addresses the flow of firearms used to commit crimes by launching multijurisdictional firearms trafficking strike forces to stop illegal gun trafficking across state lines.

Guns are easily transportable, and there are relatively few barriers to gun migration and gun trafficking from states with weak gun regulations to states with strong gun regulations. For example, crime gun tracing data suggest that, in high-regulation states, crime guns are more likely to have been purchased out of state.^3,4,5^ Although actual firearm migration is difficult to measure, there is a growing body of research on interstate differences in gun regulations and within-state firearm-related violence.^6,7,8,9^ A recent study by Liu et al^9^ used data on all states from 2000 to 2017 and found that weaker firearm laws in neighboring states were associated with higher within-state firearm-related deaths and, further, that failing to account for weaker laws in neighboring states led to underestimation of the impact of the state’s own laws on within-state firearm-related deaths.

The present study extends the work of Liu et al^9^ in 2 critical ways. First, instead of considering the laws in neighboring states only, we used a spatial analysis approach that considers the geographical distance between all contiguous states in the US . Second, instead of aggregating across firearm laws, this study used a more granular approach toward categorizing firearm laws to better inform policy makers on which laws were most strongly associated with firearm-related deaths.

## Methods

### Study Sample

All states of the US were included in this cross-sectional study except Alaska and Hawaii because they are noncontiguous with other US states. The District of Columbia was excluded because it has no relevant entries on state laws in the State Firearm Law Database. The final analysis included the remaining 48 states. The study period was from January 1, 2000, to December 31, 2019. This study was deemed exempt from human participant review by the institutional review board of the University of Alabama at Birmingham. The study followed the Strengthening the Reporting of Observational Studies in Epidemiology (STROBE) reporting guideline.

### Measures

The total numbers of firearm-related deaths by state from 2000 to 2019 were extracted from the Web-based Injury Statistics Query and Reporting System (WISQARS) of the Centers for Disease Control and Prevention^10^ as main outcome variables, including deaths due to all intent, homicide excluding legal intervention, and suicide. WISQARS is a public-access, free online database that provides data on fatal and nonfatal injuries of various causes, violent death, and cost of injuries from a variety of trusted sources.

#### Outcome Measures

The rates of total firearm-related deaths of each state and year were calculated as the main outcome variable. The rates of firearm-related homicide and suicide were also calculated as secondary outcomes.

Information on state firearm laws was obtained from the State Firearm Law Database^11^ developed by Siegel et al.^12^ This database tracks the presence of 134 laws in 14 categories across all 50 states from 1991 to 2020. Categories include buyer regulations, dealer regulations, background checks, prohibition of gun purchase and possession, domestic violence–related gun laws, “stand your ground” laws, concealed carry permitting laws, assault weapons regulations, gun trafficking laws, and restrictions on places where guns may be carried. Each of the 134 laws are coded based on their year of implementation as being either present (1) or absent (0) for each state during each year. The deciles of the total number of all firearm laws were used as an index to capture the strictness of states’ own firearm regulations.

#### Firearm Regulations

For this study, the following categories of laws were identified based on previous literature as having potential association with interstate movement of firearms and firearm-related mortality^6,7,9,13,14,15,16,17^: (1) requiring universal background checks at the point of purchase for all firearms (background check laws); (2) requiring background checks for handgun sale at gun shows (gun show laws); (3) requiring a license or permit to purchase all firearms (permit laws); (4) requiring a state dealer license for sale of handguns (licensed dealer laws); (5) requiring all private sellers and licensed dealers to keep and retain records of handgun sales (record-keeping laws); (6) prohibiting any person from purchasing a handgun on behalf of another person (straw purchase laws); (7) prohibiting firearm possession for people who have committed a violent misdemeanor (violence prohibition laws); (8) requiring people to relinquish their firearms after they become prohibited from possessing them (relinquishment laws); and (9) providing authorities with discretion in deciding whether to grant a concealed carry permit, or the law bans all concealed weapons (may-issue laws).

#### Other Covariates

We controlled for additional state-level and potentially time-varying covariates that may be associated with the number of violent deaths based on previous literature.^7,9,13,18^ These covariates included population size, proportion 65 years or older, race and ethnicity (extracted from US Census Bureau American Community Survey basic race alone table^19^), unemployment rate, poverty rate, and proportion of population 25 years or older without a high school diploma, which were obtained from the US Census Bureau for 2000 to 2019.^19^ Property crime rates were obtained from the Federal Bureau of Investigation’s summary reporting system via Crime Data Explorer^20^ as a measure of the propensity for crimes in the state. The per capita number of licensed gun dealers, obtained from the US Bureau of Alcohol, Tobacco, Firearms and Explosives,^21^ and the percentage of the state’s population holding a hunting license, obtained from the National Survey of Fishing, Hunting, and Wildlife-Associated Recreation,^22^ were included as indicators of household gun ownership.^13,23,24^ As a measure of the general state sentiment toward firearm regulation, the vote share differences between the Republican and Democratic presidential candidates in each presidential election year within the study period^25^ were included, and extrapolated for years between presidential elections.

### Statistical Analysis

To investigate the association between states’ firearm laws and other states’ firearm-related deaths (interstate association), we built a spatial autoregressive linear model (spatial Durbin model), accounting for geographically correlated dependent and independent variables^26^:

y_nt_ = ρWy_nt_ + X_nt_β + P_nt_γ + WP_nt_θ + μ_n_ + τ_t_ + ϵ_nt_

where y denotes the rate of firearm-related deaths; ρ, the spatial autoregressive coefficient; W, the spatial weight matrix; X, the state-level variables, including the deciles of the total number of firearm laws and other state-level covariates; P, the laws of interest; μ, the state fixed effect; and τ, the year fixed effect. In this spatial Durbin model, coefficients corresponding to the independent variables are difficult to interpret and, moreover, do not directly correspond to obvious measures of association. To resolve this, an overall association via a total effect is then calculated^26,27^ that compares what would happen in the (hypothetical) scenario in which all those 48 contiguous states have that particular law with the (also hypothetical) scenario in which no state has that law. Furthermore, this total effect can be decomposed into within-state (direct effect) and interstate (indirect effect or spillover effect) associations.^27^ Details are shown in eMethods and eFigure 1 in the Supplement.

To account for the distance decay of the association between firearm laws and outcome, the inverse distance squared weight matrix was used for the primary analysis, because the contiguity matrix may underestimate the association. First, a basic model without any of the 9 laws of interest was built (ie, equation 1) with *P* = 0. Then each law of interest was added to the basic model one at a time, and we selected laws that resulted both in a decreased Akaike information criterion and *P* < .10 for the likelihood ratio test between the model including this law and the basic model for the final model. Then we built a pairwise correlation matrix for the selected laws to check for the potential collinearity, and highly collinear variables were removed from the final model. The final model was also used for analyzing the secondary outcomes.

We performed the following sensitivity analyses: (1) using state random effects in addition to year and census division^28^ fixed effects, (2) using a contiguity matrix instead of an inverse distance squared matrix, and (3) adding a 1-year lagged total firearm-related death term to build a dynamic spatial Durbin model and address potential temporal autocorrelation of firearm-related deaths within state.

For all models, the effect sizes (the number of deaths per 100 000 population) with 95% CIs of the within-state, interstate, and overall associations between firearm laws and firearm-related deaths were reported. Standard errors were clustered within each state. Statistical significance was set at 2-sided *P* < .05. Stata, version 16.1 (StataCorp LLC) was used for all analyses. We built spatial Durbin models using the xsmle package developed by Belotti et al,^29^ which had been used for spatial analysis in the previous literature.^30,31^

## Results

Baseline variables are summarized in Table 1. We identified 662 883 firearm-related deaths of all intents, which translated to a rate of 10.80 firearm-related deaths per 100 000 population during the study period. For this period, the median number of implemented firearm laws was 15 (range, 1-111). The distribution of how many of the 9 laws of interest were implemented for each state and the rates of firearm-related deaths of all intents in that state compared with the national median are shown for the first and last year of the study period in the Figure.

**Table 1.** State Characteristics From 2000 to 2019^a^

State characteristic	Sample, median (IQR)			*P* value^b^
	Pooled	2000	2019	
Death rate per 100 000 population				
Total firearm-related death	11.47 (8.94 to 14.45)	10.45 (7.94 to 13.35)	13.05 (10.76 to 16.35)	<.001
Firearm-related homicide	3.61 (1.90 to 5.00)	3.45 (1.94 to 5.23)	3.92 (1.82 to 6.22)	.62
Firearm-related suicide	7.65 (5.81 to 9.69)	7.03 (5.35 to 8.47)	9.52 (7.41 to 11.06)	<.001
Total No. of firearm laws	15.50 (10.00 to 29.00)	15.00 (10.50 to 24.00)	19.50 (9.50 to 39.50)	.75
Population 65 y or older, %	13.40 (12.40 to 14.70)	12.75 (11.55 to 13.65)	16.05 (15.35 to 16.84)	<.001
White population, %^c^	84.93 (77.45 to 90.51)	86.99 (79.63 to 91.94)	80.99 (71.00 to 88.90)	<.001
Poverty rate, %	13.05 (11.00 to 15.60)	11.80 (9.00 to 13.35)	12.85 (10.66 to 14.55)	<.001
Unemployment rate,	6.20 (5.00 to 7.50)	3.90 (3.10 to 4.40)	5.20 (4.20 to 5.75)	<.001
Population aged ≥25 y without high school diploma, %	12.70 (10.20 to 15.60)	14.10 (11.85 to 17.65)	10.03 (8.42 to 12.86)	<.001
Property crime, cases per 100 population	2.84 (2.31 to 3.51)	3.61 (2.89 to 4.12)	2.07 (1.55 to 2.53)	<.001
Hunting license holder, %	6.57 (3.18 to 10.69)	7.04 (3.73 to 10.93)	7.07 (3.03 to 10.12)	.17
Licensed gun dealer, No. per 100 000 residents	22.05 (14.85 to 33.60)	28.94 (20.76 to 41.66)	20.85 (14.91 to 31.62)	<.001
Vote share differences between the Republican and the Democratic presidential candidates, %^d^	2.18 (−10.33 to 18.34)	3.71 (−5.12 to 15.79)	0.56 (−14.72 to 18.83)^e^	.63

^a^ Test was performed for the whole study period.

^b^ Calculated as rank-sum test for trend.

^c^ Extracted from US Census Bureau American Community Survey basic race alone table.

^d^ Uses original data (without interpolation).

^e^ Uses 2020 data.

**Figure.**
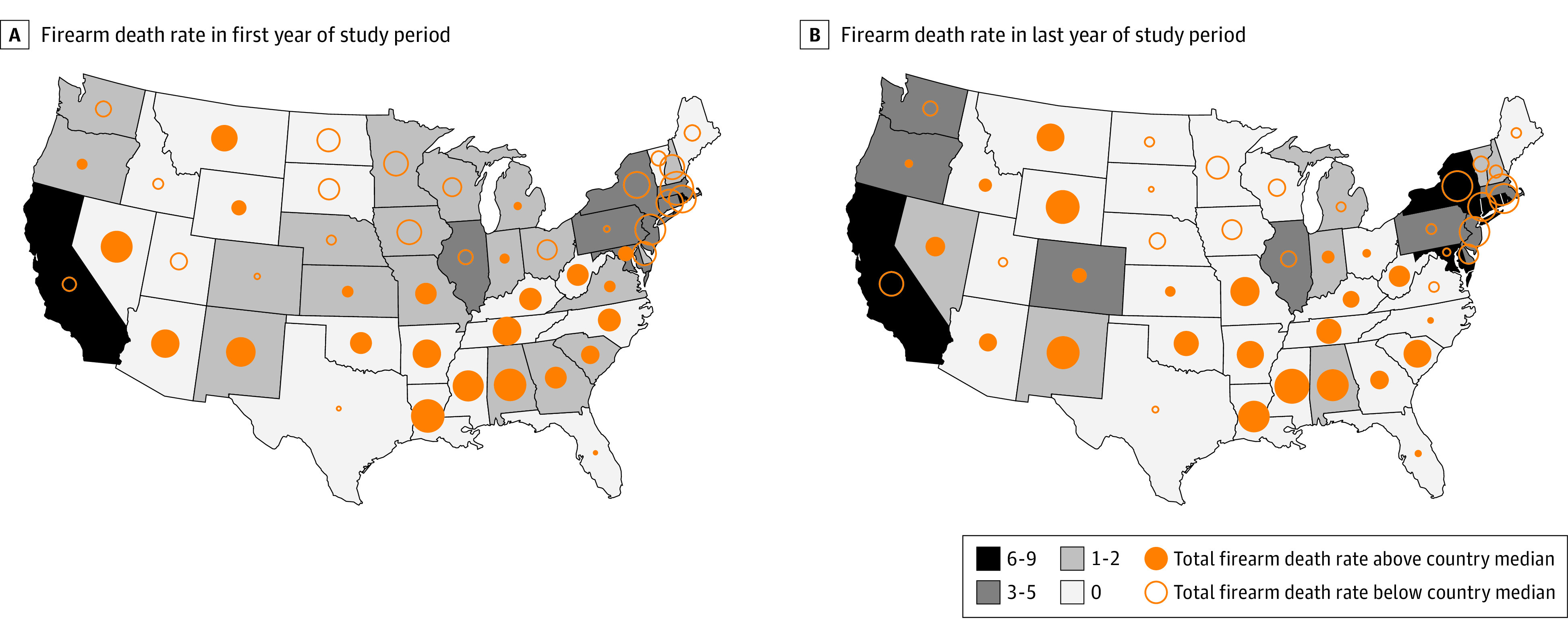
Rates of Firearm-Related Deaths of All Intents in the 48 Contiguous States Compared With the National Median for the First and Last Years of the Study Period

The effect sizes of within-state, interstate, and overall associations between each law and total firearm-related deaths rate from models where those laws were included individually are shown in eTable 1 in the Supplement. The permit (effect size, −16.72 [95% CI, −25.87 to −7.58]), record-keeping (effect size, −8.54 [955 CI, −15.49 to −1.59]), and violence prohibition (effect size, −8.55 [95% CI, −15.34 to −1.75]) laws showed interstate association with decreased firearm-related deaths in adjacent states. Permit (effect size, −2.22 [95% CI, −3.35 to −1.49]) and violence prohibition (effect size, −1.37 [95% CI, −2.07 to −0.68]) laws also showed within-state association. Permit (effect size, −19.14 [95% CI, −28.82 to −9.46]) and violence prohibition (effect size, −9.92 [95% CI, −17.11 to −2.73]) laws, in addition to the record-keeping (effect size, −9.27 [95% CI, −16.12 to −2.43]) and may-issue (effect size, −5.53 [95% CI, −10.76 to −0.29]) laws, were also associated with a decreased total firearm-related death rate.

The following 5 laws were included in the final model: (1) permit, (2) gun show, (3) violence prohibition, (4) relinquishment, and (5) may-issue (eTables 2-4 in the Supplement). As shown in Table 2, on average, the permit laws had within-state association (effect size, −1.79 [95% CI, −2.73 to −0.84]), interstate association (effect size, −10.60 [95% CI, −17.63 to −3.56]), and overall association (effect size, −12.38 [95% CI, −19.93 to −4.83]) per 100 000 population decrease in total firearm-related deaths. The violence prohibition laws had within-state association (effect size, −0.75 [95% CI, −1.37 to −0.12]) and overall association (effect size, −5.28 [95% CI, −10.33 to −0.24]) per 100 000 population decrease in total firearm-related death rate. The interstate association between this law and total firearm-related death rate showed no statistical significance (effect size, −4.54 [95% CI, −9.29 to 0.22] per 100 000 population; *P* = .06). The effect sizes of the associations reflect the differences of the outcome in the hypothetical scenario when all 48 contiguous states have that law vs none of those states have that law. eFigures 2 and 3 in the Supplement provide illustrative examples of the effect size when selected groups of states adopt the law. The results for sensitivity analyses are listed in eTables 5 to 7 in the Supplement, which were substantively similar to findings from the primary analysis.

**Table 2.** Effect Sizes of Within-State, Interstate, and Overall Association Among State Firearm Laws, State Covariables, and Total Firearm-Related Death Rates^a^

State covariable	Within-state association			Interstate association			Overall association	
	Effect size (95% CI)	*P* value	Effect size (95% CI)		*P* value	Effect size (95% CI)		*P* value
State firearm laws								
Permit	−1.79 (−2.73 to −0.84)	<.001	−10.60 (−17.63 to −3.56)		.003	−12.38 (−19.93 to −4.83)		.001
Relinquishment	−0.81 (−1.90 to 0.29)	.15	5.96 (−1.72 to 13.63)		.13	5.15 (−3.07 to 13.37)		.22
Violence prohibition	−0.75 (−1.37 to −0.12)	.02	−4.54 (−9.29 to 0.22)		.06	−5.28 (−10.33 to −0.24)		.04
Gun show	0.15 (−0.52 to 0.82)	.66	−3.23 (−7.92 to 1.46)		.18	−3.08 (−7.88 to 1.72)		.21
May-issue	−0.29 (−0.82 to 0.24)	.28	−0.36 (−4.27 to 3.55)		.86	−0.65 (−4.37 to 3.07)		.73
Index of strictness of firearm regulation^b^	−0.10 (−0.26 to 0.06)	.21	0.01 (−0.02 to 0.04)		.47	−0.09 (−0.23 to 0.05)		.22
State characteristics, %								
Population 65 y or older	0.29 (−0.01 to 0.59)	.06	−0.03 (−0.09 to 0.04)		.40	0.26 (−0.02 to 0.54)		.07
Unemployment rate	−0.08 (−0.28 to 0.11)	.41	0.01 (−0.02 to 0.04)		.56	−0.07 (−0.25 to 0.10)		.41
Cases of property crime per 100 population	1.00 (0.56 to 1.44)	<.001	−0.10 (−0.29 to 0.09)		.29	0.90 (0.49 to 1.31)		<.001
Poverty rate	−0.03 (−0.13 to 0.07)	.58	0.00 (−0.01 to 0.02)		.75	−0.03 (−0.12 to 0.07)		.58
White population^c^	0.18 (−0.01 to 0.37)	.06	−0.02 (−0.06 to 0.02)		.38	0.16 (−0.01 to 0.33)		.06
Population aged ≥25 y without high school diploma	0.05 (−0.08 to 0.18)	.46	−0.01 (−0.03 to 0.01)		.59	0.04 (−0.07 to 0.16)		.47
Hunting license holder	0.11 (0.01 to 0.20)	.03	−0.01 (−0.04 to 0.01)		.36	0.09 (0.01 to 0.18)		.03
Licensed gun dealer per 100 000 residents	−0.05 (−0.09 to −0.00)	.04	1.00 (−0.01 to 0.01)		.39	−0.04 (−0.09 to 0.00)		.06
Population density, log-transformed, person per square mile	1.41 (−3.00 to 5.82)	.53	−0.15 (−0.80 to 0.50)		.65	1.26 (−2.73 to 5.25)		.54
Vote share differences between the Republican and the Democratic presidential candidates, 10%	0.35 (0.10 to 0.59)	.005	−0.03 (−0.10 to 0.03)		.33	0.31 (0.08 to 0.54)		.008

^a^ Effect sizes are shown as per 100 000 population.

^b^ Expressed as the decile of the total number of state firearm laws.

^c^ Extracted from US Census Bureau American Community Survey basic race alone table.

For the secondary outcomes, 5 states (New Hampshire, Vermont, South Dakota, North Dakota, and Wyoming), all of which had 50% or more observations missing, were removed from the analysis of firearm homicide (additional details are provided in the eMethods in the Supplement). The within-state, interstate, and overall associations among the laws of interest, the index for the general strictness of firearm regulation, and firearm-related homicide and suicide are shown in Table 3. The permit laws had within-state association (−1.26 [95% CI, −1.72 to −0.80]), interstate association (−9.01 [95% CI, −15.00 to −3.02]), and overall association (−10.27 [95% CI, −16.53 to −4.01]) per 100 000 population decrease in firearm-related homicide rate. For firearm-related suicide, the violence prohibition laws had within-state association (effect size, −0.86 [95% CI, −1.23 to −0.50]), interstate association (effect size, −5.75 [95% CI, −8.27 to −3.22]), and overall association with (effect size, −6.61 [95% CI, −9.28 to −3.95]) per 100 000 population decrease in firearm-related suicide rate.

**Table 3.** Effect Sizes of Within-State, Interstate, and Overall Associations Between State Firearm Laws and Firearm-Related Homicide and Suicide Rates^a^^,^^b^

Firearm law	Within-state association		Interstate association		Overall association	
	Effect size (95% CI)	*P* value	Effect size (95% CI)	*P* value	Effect size (95% CI)	*P* value
**Firearm-related homicide**						
State firearm laws						
Permit	−1.26 (−1.72 to −0.80)	<.001	−9.01 (−15.00 to −3.02)	.003	−10.27 (−16.53 to −4.01)	.001
Relinquishment	−0.38 (−1.07 to 0.32)	.29	1.75 (−3.80 to 7.30)	.54	1.37 (−4.51 to 7.26)	.65
Violence prohibition	0.11 (−0.33 to 0.55)	.63	1.41 (−2.71 to 5.53)	.50	1.52 (−2.84 to 5.87)	.49
Gun show	0.06 (−0.47 to 0.60)	.82	−4.75 (−9.81 to 0.31)	.07	−4.69 (−9.91 to 0.53)	.08
May-issue	−0.29 (−0.66 to 0.07)	.12	−1.29 (−4.63 to 2.06)	.45	−1.58 (−4.82 to 1.65)	.34
Index of strictness of firearm regulation^c^	−0.03 (−0.17 to 0.12)	.72	0.00 (−0.03 to 0.03)	.89	−0.03 (−0.19 to 0.13)	.72
**Firearm-related suicide**						
State firearm laws						
Permit	−0.52 (−1.23 to 0.19)	.15	−1.10 (−5.71 to 3.51)	.64	−1.62 (−6.54 to 3.29)	.52
Relinquishment	−0.44 (−0.94 to 0.06)	.08	3.02 (−0.37 to 6.41)	.08	2.58 (−1.10 to 6.26)	.17
Violence prohibition	−0.86 (−1.23 to −0.50)	<.001	−5.75 (−8.27 to −3.22)	<.001	−6.61 (−9.28 to −3.95)	<.001
Gun show	0.03 (−0.27 to 0.32)	.85	0.91 (−1.86 to 3.69)	.52	0.94 (−1.85 to 3.74)	.51
May-issue	0.02 (−0.27 to 0.32)	.87	0.82 (−1.19 to 2.83)	.42	0.85 (−1.07 to 2.76)	.39
Index of strictness of firearm regulation^c^	−0.07 (−0.14 to 0.00)	.06	0.01 (−0.01 to 0.03)	.23	−0.06 (−0.12 to 0.00)	.07

^a^ Effect sizes are shown as per 100 000 population.

^b^ Models were adjusted for the following state-level time-varying variables: population size, proportion 65 years or older, race and ethnicity, unemployment rate, poverty rate, proportion 25 years or older without a high school diploma, the rates of crime against property, the percentage of state’s population holding a hunting license, number of licensed gun dealers per 100 000 residents, and the vote share difference between the Republican and Democratic candidates in the presidential election (linearly interpolated), in addition to state and year fixed effect.

^c^ Expressed as the decile of the total number of state firearm laws.

## Discussion

In this cross-sectional study, we used data from 2000 to 2019 to investigate the association between firearm laws and firearm-related deaths both within state and interstate. Our findings suggest that having a permit requirement for purchasing all firearms was associated with a decrease of any firearm-related death and firearm-related homicide within state, interstate, and overall. The prohibition of firearm possession for individuals who have committed a violent misdemeanor had within-state and overall associations with decreased total firearm-related death rates. This law also had within-state, interstate, and overall associations with decreased firearm-related suicide rates.

Firearm laws vary substantially across states in the US. A rich literature has established associations between stricter state firearm laws and reduced firearm-related violence within those states.^17,32,33,34^ States with strict laws also host fewer firearm-manufacturing establishments than states with relatively permissive laws.^35^ However, firearms are easily transported across state lines, and crime guns in states with restrictive firearm laws often originate in states with more permissive rules,^3,5^ with stronger trafficking flows detected when such states are near each other.^4,36^ Hence, a small but growing literature^6,7,8,9^ has started exploring whether permissive firearm laws in one state have spillover effects on measures of firearm violence in other states. More recently, Morrison et al^36^ used county-level firearm-related homicide data to evaluate the spillover effects of different categories of firearm laws. They found that the benefits of within-state firearm laws in reducing firearm-related homicide were not independent of firearm laws in nearby states, which is supported by our study findings.

Our study adds to this literature by evaluating specific firearm laws individually rather than as an aggregated index. Particularly, our findings suggest that permit-to-purchase laws, which research finds to be protective against within-state firearm-related deaths,^17^ are also protective for neighboring states, suggesting that more restrictions on the eligibility of obtaining a gun also discourage out-of-state persons from buying firearms from those states. Further, the laws prohibiting firearm possession for people who have committed a violent misdemeanor were associated with decreased firearm-related suicide rates within state, interstate, and overall and with decreased total firearm-related death rates both within state and overall. Prior studies have found that safe storage laws^37^ and alcohol regulation^38^ were associated with within-state declines in firearm suicides. At the same time, other studies^39,40^ have established correlations between committing acts of violence and inflicting self-harm,^39,40^ and our findings imply that regulations preventing firearm possession among those who commit violence in turn also reduce extreme self-harm in both within-state and nearby state populations. We speculate that spillover protective effects occur because the presence of these laws disincentivizes gun traffickers from acquiring guns in these states to send to neighboring states and disincentivizes individuals in neighboring states from crossing state borders and purchasing a weapon.

In models that included laws individually with other state covariates, may-issue and record-keeping laws also showed associations with total firearm-related deaths. However, collinearity issues (eTable 3 in the Supplement) and a tendency among states to concurrently implement regulations regarding gun show, background check, and record-keeping laws (eTables 8 and 9 in the Supplement) limited our ability to include them in the final model. Notably, some of the firearm laws that were intended to reduce firearm-related deaths did not show any conclusive association. This could be either a true nonassociation or an association that could not be detected by our method owing to limited sample size, rarity of states that have such laws, and insufficient time variability of those laws during our study period.

### Limitations

We acknowledge several limitations to this study. First, our analytical strategy used state fixed effects to minimize the effect of time-invariant state-level confounders and hence relied on within-state variation in firearm laws to estimate effect sizes; however, many states had minimal variations in firearm laws during the study period, and states implementing laws such as background check and record keeping often implemented them at similar times, leading to collinearity issues. These factors may have limited the ability of our model to detect statistical effects for certain laws. Second, the presence of laws per se may not indicate the diligence with which laws are enforced in different states. Third, results may be sensitive to alternate approaches to selecting or grouping firearm laws of primary interest. Fourth, the effect sizes can be affected by the matrix accounting for the geographic correlation. Moreover, the weight matrix used in this study solely focused on the distance. Combination of distance with other factors, such as population, firearm manufacturing facilities, and number of interstates and other highways, might be more accurate to describe the attractiveness of firearms for a state and the ease of firearm movement between states. Fifth, because the analysis of firearm-related homicide did not use data from all states, caution should be used when generalizing the findings to the whole country. Last, as in the case of all ecological studies, caution should be exercised when drawing causal inferences from the results.

## Conclusions

In this pooled cross-sectional analysis of firearm laws and firearm-related deaths from 2000 to 2019, we found that permit-to-purchase laws were associated with decreased firearm-related death rates both within state and interstate. The presence of interstate association between firearm laws and firearm-related deaths suggests that policy initiatives to reduce gun trafficking—such as those adopted by the Biden-Harris administration—are an important component of eliminating firearm violence. It also underlines the importance of synergic legislative action to implement laws such as permit requirements in proximate states as an effective approach to reduce firearm-related deaths.

## Notes

Supplement. **eMethods.** Statistical Analysis **eFigure 1.** Spatial Autocorrelations for the Numbers of Firearm Laws of Interest Implemented and Total Firearm-Related Deaths **eTable 1.** Effect Sizes of Within-State, Interstate, and Overall Association Between Laws of Interest and Total Firearm-Related Death Rate From the Spatial Durbin Model (SDM) **eTable 2.** Variable Selection Based on Akaike Information Criterion (AIC) and Likelihood Ratio Test **eTable 3.** Pairwise Correlation Matrix for All 9 Laws of Interest **eTable 4.** Effect Sizes of Within-State, Interstate, and Overall Association Among Laws of Interest, the Index of Strictness of Firearm Regulation, State’s Covariables, and Total Firearm-Related Death Rates **eFigure 2.** Geographical Distribution of the Decrease of Predicted Total Effect of Total Firearm-Related Death Rate in 2019 of Each State if Alabama Hypothetically Had Had Permit-to-Purchase Law Implemented in 2019 **eFigure 3.** Decrease of Predicted Total Effect of Total Firearm-Related Death Rate in 2019 if All South-West Central States Had Had Permit-to-Purchase Law Implemented in 2019 Hypothetically **eTable 5.** State Random-Effect SDM **eTable 6.** SDM Using Contiguity Weight Matrix, Otherwise Same as the Final Model **eTable 7.** Short- and Long-term Associations Among Laws of Interest, Index of Strictness of Firearm Regulation, and Total Firearm-Related Death Rates From a Dynamic Spatial Durbin Model Including a 1-Year Lagged Outcome Variable in the Model **eTable 8.** Years of Implementation of 3 Selected Laws (2000-2019) **eTable 9.** Number of States That Have Implemented the 9 Laws of Interest
