# Supplementary material for: Association of State-Level Firearm-Related Deaths With Firearm Laws in Neighboring States
Source: JAMA Netw Open. 2022 Nov 8;5(11):e2240750. doi: 10.1001/jamanetworkopen.2022.40750 (PMC9644258; doi:10.1001/jamanetworkopen.2022.40750)
Supplement: Supplement. — eMethods. Statistical Analysis eFigure 1. Spatial Autocorrelations for the Numbers of Firearm Laws of Interest Implemented and Total Firearm-Related Deaths eTable 1. Effect Sizes of Within-State, Interstate, and Overall Association Between Laws of Interest and Total Firearm-Related Death Rate From the Spatial Durbin Model (SDM) eTable 2. Variable Selection Based on Akaike Information Criterion (AIC) and Likelihood Ratio Test eTable 3. Pairwise Correlation Matrix for All 9 Laws of Interest eTable 4. Effect Sizes of Within-State, Interstate, and Overall Association Among Laws of Interest, the Index of Strictness of Firearm Regulation, State’s Covariables, and Total Firearm-Related Death Rates eFigure 2. Geographical Distribution of the Decrease of Predicted Total Effect of Total Firearm-Related Death Rate in 2019 of Each State if Alabama Hypothetically Had Had Permit-to-Purchase Law Implemented in 2019 eFigure 3. Decrease of Predicted Total Effect of Total Firearm-Related Death Rate in 2019 if All South-West Central States Had Had Permit-to-Purchase Law Implemented in 2019 Hypothetically eTable 5. State Random-Effect SDM eTable 6. SDM Using Contiguity Weight Matrix, Otherwise Same as the Final Model eTable 7. Short- and Long-term Associations Among Laws of Interest, Index of Strictness of Firearm Regulation, and Total Firearm-Related Death Rates From a Dynamic Spatial Durbin Model Including a 1-Year Lagged Outcome Variable in the Model eTable 8. Years of Implementation of 3 Selected Laws (2000-2019) eTable 9. Number of States That Have Implemented the 9 Laws of Interest [file jamanetwopen-e2240750-s001.pdf]

Supplementary Online Content

Liu Y, Siegel M, Sen B. Association of state-level firearm-related deaths with firearm laws in neighboring states. *JAMA Netw Open*. 2022;5(11):e2240750.
doi:10.1001/jamanetworkopen.2022.40750

eMethods. Statistical Analysis
eFigure 1. Spatial Autocorrelations for the Numbers of Firearm Laws of Interest Implemented and Total Firearm-Related Deaths
eTable 1. Effect Sizes of Within-State, Interstate, and Overall Association Between Laws of Interest and Total Firearm-Related Death Rate From the Spatial Durbin Model (SDM)
eTable 2. Variable Selection Based on Akaike Information Criterion (AIC) and Likelihood Ratio Test
eTable 3. Pairwise Correlation Matrix for All 9 Laws of Interest
eTable 4. Effect Sizes of Within-State, Interstate, and Overall Association Among Laws of Interest, the Index of Strictness of Firearm Regulation, State's Covariables, and Total Firearm-Related Death Rates
eFigure 2. Geographical Distribution of the Decrease of Predicted Total Effect of Total Firearm-Related Death Rate in 2019 of Each State if Alabama Hypothetically Had Had Permit-to-Purchase Law Implemented in 2019
eFigure 3. Decrease of Predicted Total Effect of Total Firearm-Related Death Rate in 2019 if All South-West Central States Had Had Permit-to-Purchase Law Implemented in 2019 Hypothetically
eTable 5. State Random-Effect SDM
eTable 6. SDM Using Contiguity Weight Matrix, Otherwise Same as the Final Model
eTable 7. Short- and Long-term Associations Among Laws of Interest, Index of Strictness of Firearm Regulation, and Total Firearm-Related Death Rates From a Dynamic Spatial Durbin Model Including a 1-Year Lagged Outcome Variable in the Model
eTable 8. Years of Implementation of 3 Selected Laws (2000-2019)
eTable 9. Number of States That Have Implemented the 9 Laws of Interest

This supplementary material has been provided by the authors to give readers additional information about their work.

© 2022 Liu Y et al. *JAMA Network Open*.

eMethods. Statistical Analysis

The spatial Durbin model (SDM) used in this analysis:

$$y_{nt} = \rho W y_{nt} + X_{nt}\beta + P_{nt}\gamma + WP_{nt}\theta + \mu_n + \tau_t + \epsilon_{nt} \qquad (1)$$

where y denotes the rate of firearm death; ρ denotes spatial autoregressive coefficient; W denotes the spatial weight matrix; X denotes the state level variables including the deciles of the total number of firearm laws and other state level covariates; P denotes the laws of interest; μ and τ denote the state and year fixed effect, respectively, where the former controls for state-specific time-invariant confounders, and the latter accounts for confounders that change from year to year for the whole country. For the spatial weight matrix W, each cell of the matrix, w_{ij}, reflects the intensity of the spatial interaction between unit i and unit j. By convention, $w_{ii} = 0$, so that the W matrix has a zero diagonal.

For the kth regressor of P, if we use $M^{\bar{d}}$ to denotes the mean diagonal element of matrix M, and the $M^{\overline{rsum}}$ to denote the mean row sum of the non-diagonal elements of matrix M, then

The direct effects (DE):

$$\mathrm{DE} = \{(\mathrm{I} - \rho \mathrm{W})^{-1} \times (\gamma_k \mathrm{I} + \theta_k \mathrm{W})\}^{\bar{d}}$$

The indirect effect (IE):

$$\mathrm{IE} = \{(\mathrm{I} - \rho \mathrm{W})^{-1} \times (\gamma_k \mathrm{I} + \theta_k \mathrm{W})\}^{\overline{rsum}}$$

The total effect (TE) is the sum of DE and IE.

See: Elhorst, J. Paul. Spatial econometrics: from cross-sectional data to spatial panels. Heidelberg: Springer, 2014.

Evaluation of the spatial correlation for the firearm laws of interests and the firearm death rate.

The total number of the firearm laws of interest was calculated and then the mean of each year was used to evaluate the spatial correlation. Moran’s index was used from describe the spatial correlation (eFigure 1). Similar method was implemented for evaluating the spatial correlation of the rate of total firearm death.

Missing values.

The “xsmle” generally cannot handle unbalanced panels. The WISQARS system suppressed the number of deaths if the counts were <10, which led to missing values for some state-years. Among our outcomes, firearm homicides (excluding legal intervention) had missing values for 8.3% of state-year observations, involving eight states. To achieve balanced panel data, for three states with relatively few observations missing (Maine with 6 missing observations; Delaware with 1 missing observation; Montana with 1 missing observation), the missing values were imputed as nine, while the remaining states (New Hampshire, Vermont, South Dakota, North Dakota, and Wyoming), all of which had 50% or more observations missing, were removed from the analysis of firearm homicide.

© 2022 Liu Y et al. *JAMA Network Open*.

eFigure 1. Spatial Autocorrelations for the Numbers of Firearm Laws of Interest Implemented and Total Firearm-Related Deaths

A) Mean of total numbers of the laws of interest for each year and the Moran's I showing positive spatial correlation. P values for all Moran's I are <0.001

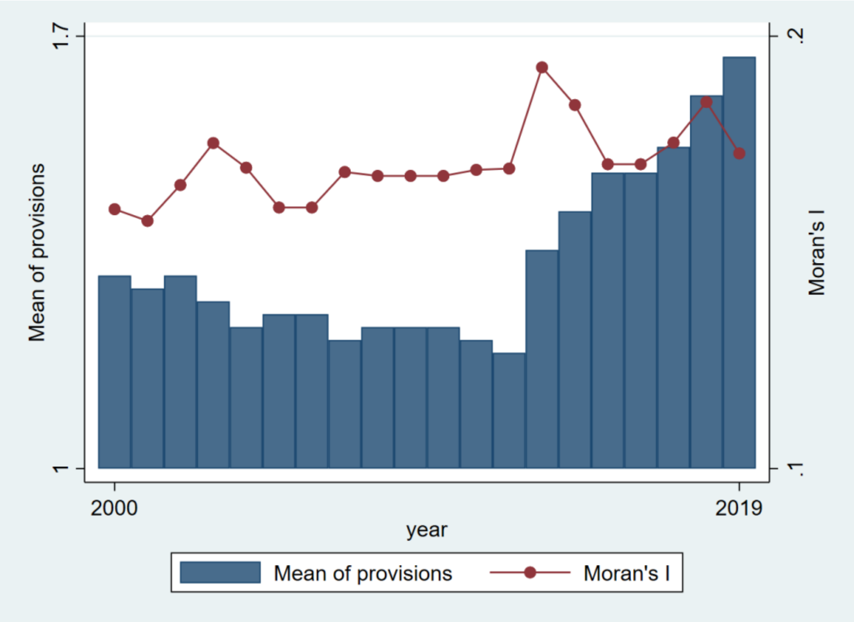

B) Mean of firearm death rate for each year and the Moran's I showing positive spatial correlation. P values for all Moran's I are <0.001

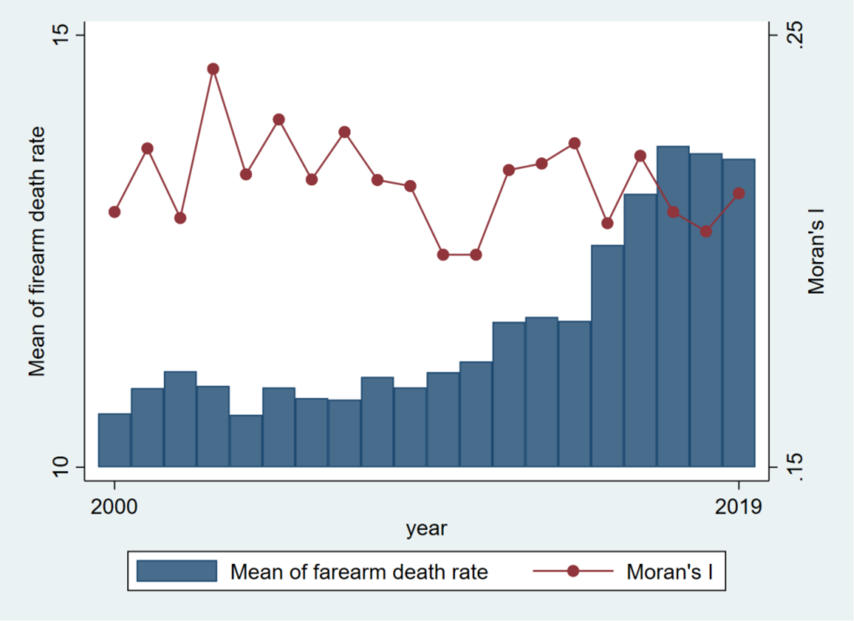

© 2022 Liu Y et al. *JAMA Network Open*.

eTable 1. Effect Sizes of Within-State, Interstate, and Overall Association Between Laws of Interest and Total Firearm-Related Death Rate From the Spatial Durbin Model (SDM)

	Within-state association			Interstate association			Overall association		
	Effect size	95% CI	P value	Effect size	95% CI	P value	Effect size	95% CI	P value
Background check	-0.28	[-1.31 - 0.74]	0.588	-6.17	[-13.37 - 1.03]	0.093	-6.45	[-13.66 - 0.76]	0.08
Gun show	0.01	[-0.68 - 0.71]	0.968	-9.36	[-18.73 - 0.00]	0.05	-9.35	[-18.94 - 0.24]	0.056
Permit	-2.42	[-3.35 - -1.49]	<0.001	-16.72	[-25.87 - -7.58]	<0.001	-19.14	[-28.82 - -9.46]	<0.001
Licensed dealer	-0.45	[-1.26 - 0.35]	0.272	-0.57	[-13.52 - 12.37]	0.931	-1.02	[-14.20 - 12.15]	0.879
Records keeping	-0.73	[-1.73 - 0.27]	0.15	-8.54	[-15.49 - -1.59]	0.016	-9.27	[-16.12 - -2.43]	0.008
Straw purchase	0.01	[-0.36 - 0.37]	0.977	-4.62	[-20.47 - 11.23]	0.568	-4.62	[-20.64 - 11.41]	0.572
May issue	-0.4	[-0.93 - 0.13]	0.135	-5.12	[-10.50 - 0.25]	0.062	-5.53	[-10.76 - -0.29]	0.038
Violence prohibition	-1.37	[-2.07 - -0.68]	<0.001	-8.55	[-15.34 - -1.75]	0.014	-9.92	[-17.11 - -2.73]	0.007
Relinquishment	-0.63	[-2.35 - 1.09]	0.471	6.16	[-9.04 - 21.36]	0.427	5.53	[-10.83 - 21.88]	0.508

*: Effect sizes are shown as per 100,000 population.

**: For all models, laws were added one at a time, with the following state-level time-varying variables—deciles of the total number of all firearm laws, population size, proportion aged ≥65 years, race, unemployment rate, poverty rate, the proportion aged ≥25 years without a high school diploma, the rates of crime against property, the percentage of state's population holding a hunting license, licensed gun dealer per 100,000 residents, and the vote share difference between the Republican and Democratic candidates in the presidential election (linear interpolated)—adjusted for, in addition to state and year fixed effect.

© 2022 Liu Y et al. *JAMA Network Open*.

eTable 2. Variable Selection Based on Akaike Information Criterion (AIC) and Likelihood Ratio Test

	AIC, one at a time	P value for LR test	AIC, stepwise‡
Basic model	2944.293		
Permit§	2595.523	0.0000	2595.523
Relinquishment§	2635.941	0.0021	2585.296
Violence prohibition§	2618.624	0.0000	2574.606
Gun show§	2633.305	0.0006	2573.409*
May issue§	2633.634	0.0007	2574.516**
Straw purchase	2647.268	0.5991	2577.127**
Records keeping†	2633.213	0.0005	2573.689**
Background check†	2641.231	0.0293	2576.732**
Licensed dealer	2645.665	0.2688	2575.268**

§: selected for the final model

†: met the criteria for AIC and LR test, but was removed for colinearity

*: Minimum AIC achieved

**: AICs of the models including basic model, 4 laws selected, and the corresponding law

‡: An alternative approach that attempted to achieve minimum AIC is used for laws selection. From the basic model, each laws of interest is added one at a time, and the one that can achieve the minimum AIC is kept. This process is repeated until the minimum AIC was achieved.

© 2022 Liu Y et al. *JAMA Network Open*.

eTable 3. Pairwise Correlation Matrix for All 9 Laws of Interest

	Background check	Gun show	Permit	Licensed dealer	Records keeping	Straw purchase	May issue	Violence prohibition	relinquishment
Background check	1								
Gun show	0.6678	1							
Permit	0.255	0.2609	1						
Licensed dealer	0.3765	0.4331	0.3437	1					
Records keeping	0.6227	0.712	0.4694	0.4691	1				
Straw purchase	0.1726	0.3507	0.2542	0.4219	0.3549	1			
May issue	0.3974	0.4255	0.5075	0.5317	0.4868	0.3738	1		
Violence prohibition	0.4224	0.391	0.142	0.2993	0.3948	0.5607	0.3448	1	
relinquishment	0.2508	0.3783	0.3731	0.3872	0.3827	0.4337	0.2602	0.128	1

© 2022 Liu Y et al. *JAMA Network Open*.

eTable 4. Effect Sizes of Within-State, Interstate, and Overall Association Among Laws of Interest, the Index of Strictness of Firearm Regulation, State's Covariables, and Total Firearm-Related Death Rates

	Within-state association			Interstate association			Overall association		
	Effect size	95% CI	P value	Effect size	95% CI	P value	Effect size	95% CI	P value
Permit*	**-1.84**	**[-2.84 - -0.85]**	**<0.001**	**-11.26**	**[-18.73 - -3.79]**	**0.003**	**-13.1**	**[-21.10 - -5.11]**	**0.001**
Relinquishment*	-0.77	[-1.93 - 0.39]	0.191	6.05	[-1.59 - 13.69]	0.121	5.28	[-2.93 - 13.49]	0.207
Violence prohibition*	**-0.81**	**[-1.41 - -0.21]**	**0.008**	**-5.3**	**[-10.15 - -0.45]**	**0.032**	**-6.11**	**[-11.20 - -1.03]**	**0.018**
Gun show*	0.19	[-0.49 - 0.87]	0.582	-3.36	[-8.42 - 1.70]	0.193	-3.17	[-8.36 - 2.01]	0.231
Index of strictness of firearm regulation (deciles of number of total firearm laws)	-0.11	[-0.26 - 0.04]	0.139	0.01	[-0.02 - 0.04]	0.503	-0.1	[-0.24 - 0.03]	0.142
Population 65 years or older (%)	0.27	[0.01 - 0.54]	0.044	-0.02	[-0.08 - 0.04]	0.502	0.25	[-0.01 - 0.51]	0.059
Unemployment rate	-0.1	[-0.31 - 0.12]	0.381	0.01	[-0.02 - 0.04]	0.62	-0.09	[-0.29 - 0.11]	0.387
Property crime (per 100 population)	0.94	[0.54 - 1.33]	<0.001	-0.08	[-0.27 - 0.11]	0.407	0.86	[0.46 - 1.25]	<0.001
Poverty rate (%)	-0.02	[-0.12 - 0.08]	0.699	0.00	[-0.01 - 0.01]	0.829	-0.02	[-0.11 - 0.08]	0.699
White population (%)	0.18	[0.00 - 0.36]	0.045	-0.02	[-0.06 - 0.03]	0.444	0.17	[0.00 - 0.33]	0.047
Population aged ≥25 years without high school diploma (%)	0.05	[-0.08 - 0.17]	0.44	0.00	[-0.02 - 0.01]	0.697	0.05	[-0.07 - 0.16]	0.446
Hunting license holder (%)	0.11	[0.01 - 0.21]	0.026	-0.01	[-0.03 - 0.02]	0.465	0.11	[0.01 - 0.20]	0.034
Licensed gun dealer (per 100,000 residents)	-0.05	[-0.09 - -0.01]	0.028	0.00	[-0.01 - 0.01]	0.503	-0.05	[-0.09 - -0.00]	0.04
Population density (logged)	1.04	[-3.70 - 5.79]	0.666	-0.08	[-0.75 - 0.58]	0.805	0.96	[-3.44 - 5.36]	0.669
Vote share differences between the Republican and the Democratic presidential candidates (10%)	0.34	[0.09 - 0.60]	0.009	-0.03	[-0.10 - 0.05]	0.476	0.32	[0.06 - 0.58]	0.016

#: Effect sizes are shown as per 100,000 population.

*: Laws are selected by a stepwise approach to achieve minimal AIC

© 2022 Liu Y et al. *JAMA Network Open*.

Illustrative examples (eFigure 2 and 3).

This is an illustrative prediction to show the decrease of the total firearm death rate (total effect) if Alabama had had "Permit-to-purchase" law implemented in 2019, with all other factors constant. The map showed the decrease in total firearm death rate in each state between the predicted values ("total effect") (permit=0 vs. permit=1 of the corresponding state in corresponding year). Colors are coded in quartiles; deeper blue indicates more decrease in predicted total firearm death rate.

eFigure 2. Geographical Distribution of the Decrease of Predicted Total Effect of Total Firearm-Related Death Rate in 2019 of Each State if Alabama Hypothetically Had Had Permit-to-Purchase Law Implemented in 2019

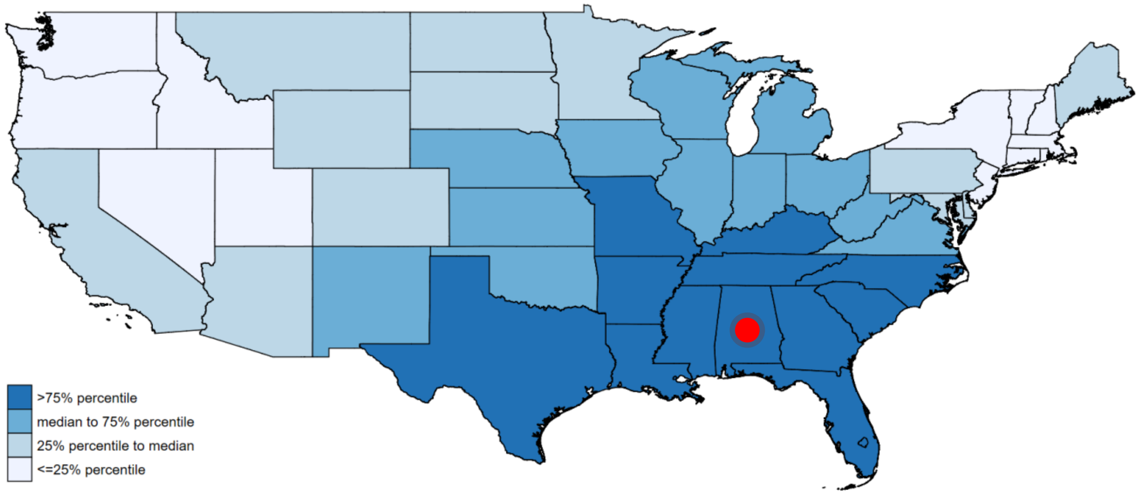

© 2022 Liu Y et al. *JAMA Network Open*.

We then repeated this prediction to show the decrease of the total firearm death rate if all South-West Central (Census division) states (TX, AR, LA, and OK) had had “Permit-to-purchase” law implemented in 2019 hyperthetically, with all other factors constant. Weighted by the inverse of the variance of the predicted rate, on average, there is a decrease of total firearm deaths (total effect) by 1.05 per 100,000 population in 2019 (95% CI: 0.16 – 1.94, p=0.022) across all 48 contiguous states.
eFigure 3 showed the differences summarized at census division level.

eFigure 3. Decrease of Predicted Total Effect of Total Firearm-Related Death Rate in 2019 if All South-West Central States Had Had Permit-to-Purchase Law Implemented in 2019 Hypothetically

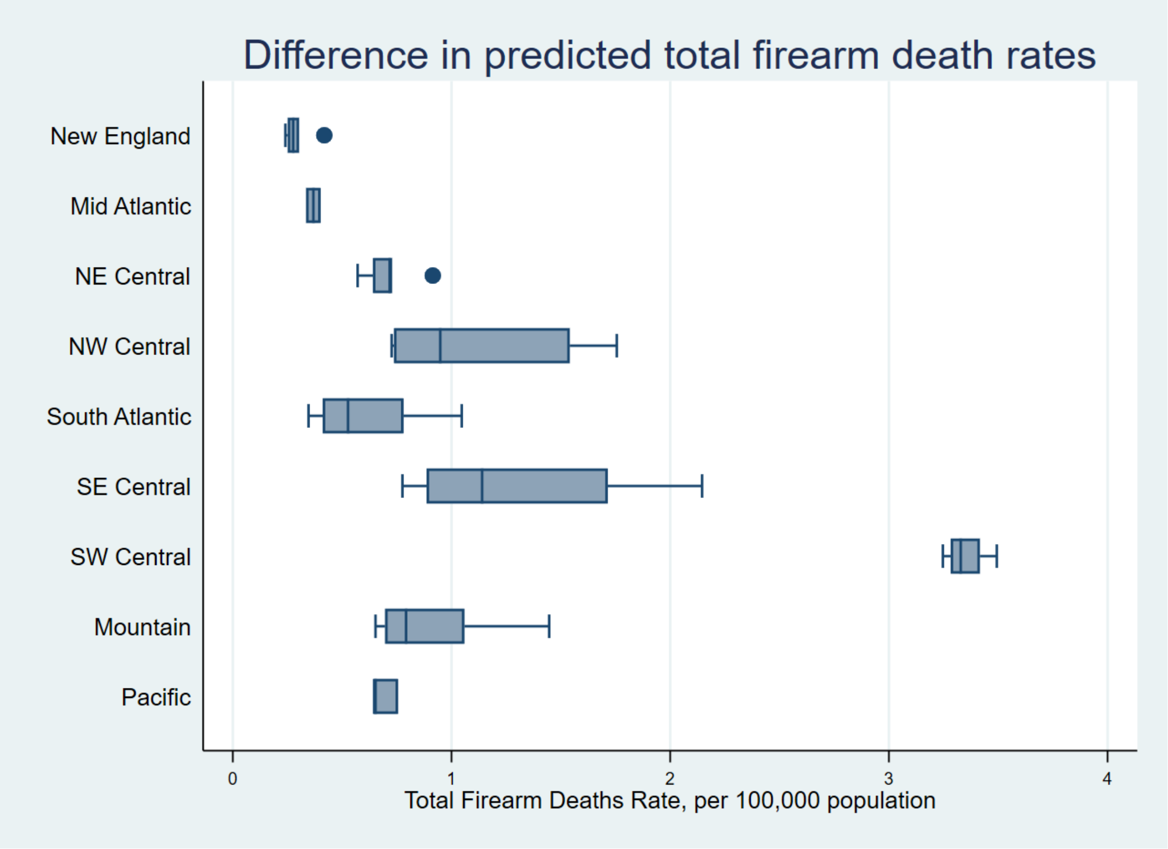

Results are summarized at the Census division level.

Sensitivity analysis (eTables 5, 6, and 7)

The following 2 tables (eTable 5 and eTable 6) illustrate two sensitivity analyzes performed for the total firearm deaths to test the robustness of the results. First, the final model was repeated but using a contiguity weight matrix instead of the original inverse distance squared weight matrix. Second, a state random effect model with year- and census division- fixed effect (9 census divisions) using the same independent variables and the same weighted independent variables as the final model was built. Effect sizes and 95% CI are shown.

© 2022 Liu Y et al. *JAMA Network Open*.

eTable 5. State Random-Effect SDM

		Effect size#	
	Within-state association	Interstate association	Overall association
Permit	-2.108***	-11.78***	-13.89***
	(-3.018 - -1.199)	(-17.24 - -6.329)	(-19.69 - -8.092)
Relinquishment	-1.056**	3.515	2.459
	(-2.032 - -0.0808)	(-3.772 - 10.80)	(-5.222 - 10.14)
Violence prohibition	-0.719*	-4.925**	-5.644**
	(-1.508 - 0.0701)	(-9.601 - -0.250)	(-10.68 - -0.609)
Gun show	0.0188	-4.231*	-4.213*
	(-0.612 - 0.649)	(-8.843 - 0.380)	(-8.948 - 0.523)
May issue	-0.256	-0.415	-0.671
	(-0.760 - 0.248)	(-4.226 - 3.395)	(-4.337 - 2.995)
Index of strictness of firearm regulation (deciles of number of total firearm laws)	-0.125	0.0119	-0.113
	(-0.294 - 0.0444)	(-0.0226 - 0.0463)	(-0.270 - 0.0439)
Population 65 years or older (%)	0.376**	-0.0339	0.342**
	(0.0813 - 0.671)	(-0.118 - 0.0497)	(0.0474 - 0.637)
Unemployment rate	-0.1	0.0105	-0.0897
	(-0.280 - 0.0798)	(-0.0216 - 0.0427)	(-0.252 - 0.0728)
Property crime (per 100 population)	1.043***	-0.106	0.937***
	(0.596 - 1.490)	(-0.318 - 0.105)	(0.512 - 1.361)
Poverty rate (%)	-0.0224	0.00142	-0.0209
	(-0.134 - 0.0897)	(-0.0135 - 0.0164)	(-0.124 - 0.0823)
White population (%)	0.0447	-0.00422	0.0404
	(-0.0980 - 0.187)	(-0.0260 - 0.0176)	(-0.0894 - 0.170)
Population aged ≥25 years without high school diploma (%)	0.0499	-0.00542	0.0445
	(-0.0755 - 0.175)	(-0.0251 - 0.0143)	(-0.0685 - 0.157)
Hunting license holder (%)	0.0828*	-0.00857	0.0742*
	(-0.0132 - 0.179)	(-0.0290 - 0.0119)	(-0.0129 - 0.161)
Licensed gun dealer (per 100,000 residents)	-0.0367	0.0032	-0.0335
	(-0.0999 - 0.0264)	(-0.00741 - 0.0138)	(-0.0925 - 0.0254)
Population density (logged)	-0.159	0.0201	-0.139
	(-1.463 - 1.144)	(-0.163 - 0.203)	(-1.325 - 1.047)
Vote share differences between the Republican and the Democratic presidential candidates (10%)	0.345***	-0.0327	0.312***
	(0.107 - 0.582)	(-0.104 - 0.0384)	(0.0791 - 0.545)

. All laws and covariates included in the final model were also included in this model, in addition to a year fixed effect and a census division fixed effect

© 2022 Liu Y et al. *JAMA Network Open*.

*** p<0.01, ** p<0.05, * p<0.1

#: Effect sizes are shown as per 100,000 population.

eTable 6. SDM Using Contiguity Weight Matrix, Otherwise Same as the Final Model

		Effect size#	
	Within-state association	Interstate association	Overall association
Permit	-1.447***	-3.792***	-5.238***
	(-2.523 - -0.370)	(-5.844 - -1.739)	(-7.982 - -2.494)
Relinquishment	-0.911*	-0.431	-1.342
	(-1.844 - 0.0212)	(-2.231 - 1.369)	(-3.749 - 1.064)
Violence prohibition	-0.752**	-1.695	-2.447*
	(-1.415 - -0.0897)	(-4.111 - 0.721)	(-5.097 - 0.202)
Gun show	0.0459	-1.427**	-1.382
	(-0.637 - 0.729)	(-2.794 - -0.0611)	(-3.052 - 0.288)
May issue	-0.337	0.0898	-0.247
	(-0.813 - 0.140)	(-0.849 - 1.028)	(-1.135 - 0.642)
Index of strictness of firearm regulation (deciles of number of total firearm laws)	-0.1	-0.00772	-0.108
	(-0.261 - 0.0613)	(-0.0247 - 0.00928)	(-0.282 - 0.0664)
Population 65 years or older (%)	0.22	0.019	0.239
	(-0.0478 - 0.488)	(-0.0147 - 0.0526)	(-0.0547 - 0.533)
Unemployment rate	-0.105	-0.0093	-0.115
	(-0.308 - 0.0969)	(-0.0314 - 0.0128)	(-0.335 - 0.106)
Property crime (per 100 population)	0.913***	0.077	0.990***
	(0.483 - 1.344)	(-0.0169 - 0.171)	(0.510 - 1.471)
Poverty rate (%)	-0.00439	-0.000736	-0.00513
	(-0.106 - 0.0970)	(-0.0104 - 0.00892)	(-0.115 - 0.105)
White population (%)	0.151*	0.0124	0.164*
	(-0.0209 - 0.324)	(-0.00827 - 0.0330)	(-0.0228 - 0.350)
Population aged ≥25 years without high school diploma (%)	0.0294	0.00265	0.0321
	(-0.105 - 0.163)	(-0.0105 - 0.0158)	(-0.114 - 0.178)
Hunting license holder (%)	0.113**	0.00977	0.123**
	(0.0197 - 0.206)	(-0.00463 - 0.0242)	(0.0195 - 0.226)
Licensed gun dealer (per 100,000 residents)	-0.0468**	-0.00382	-0.0506**
	(-0.0932 - -0.000345)	(-0.00969 - 0.00206)	(-0.101 - -0.000443)
Population density (logged)	1.099	0.0889	1.188
	(-3.062 - 5.261)	(-0.304 - 0.482)	(-3.319 - 5.695)

© 2022 Liu Y et al. *JAMA Network Open*.

Vote share differences between the Republican and the Democratic presidential candidates (10%)	0.411***	0.0346	0.445***
	(0.137 - 0.684)	(-0.0104 - 0.0797)	(0.144 - 0.746)

*** p<0.01, ** p<0.05, * p<0.1

#: Effect sizes are shown as per 100,000 population.

© 2022 Liu Y et al. *JAMA Network Open*.

eTable 7. Short- and Long-term Associations Among Laws of Interest, Index of Strictness of Firearm Regulation, and Total Firearm-Related Death Rates From a Dynamic Spatial Durbin Model Including a 1-Year Lagged Outcome Variable in the Model

	Within-state association			Interstate association			Overall association		
	Effect size*	95% CI	P value	Effect size*	95% CI	P value	Effect size*	95% CI	P value
Short term									
Permit	**-1.22**	**[-1.88 - -0.56]**	**<0.001**	**-6.3**	**[-10.90 - -1.70]**	**0.007**	**-7.52**	**[-12.46 - -2.57]**	**0.003**
Relinquishment	-0.51	[-1.25 - 0.22]	0.169	4.81	[-0.76 - 10.39]	0.091	4.3	[-1.68 - 10.29]	0.159
Violence prohibition	**-0.58**	**[-1.04 - -0.13]**	**0.012**	**-4.3**	**[-7.20 - -1.40]**	**0.004**	**-4.88**	**[-8.03 - -1.73]**	**0.002**
Gun show	0.03	[-0.36 - 0.42]	0.889	-1.1	[-4.08 - 1.88]	0.468	-1.07	[-4.19 - 2.04]	0.499
May issue	-0.26	[-0.69 - 0.18]	0.248	-0.37	[-2.83 - 2.10]	0.77	-0.62	[-2.95 - 1.70]	0.598
Index of strictness of firearm regulation (deciles of number of total firearm laws)	-0.07	[-0.18 - 0.04]	0.186	0.01	[-0.01 - 0.04]	0.315	-0.06	[-0.16 - 0.03]	0.198
Long term									
Permit	**-2.09**	**[-3.25 - -0.94]**	**<0.001**	**-9.77**	**[-17.87 - -1.67]**	**0.018**	**-11.86**	**[-20.53 - -3.19]**	**0.007**
Relinquishment	-0.95	[-2.22 - 0.33]	0.145	7.73	[-1.31 - 16.77]	0.094	6.79	[-2.93 - 16.50]	0.171
Violence prohibition	**-0.99**	**[-1.77 - -0.21]**	**0.013**	**-6.62**	**[-11.18 - -2.06]**	**0.004**	**-7.61**	**[-12.54 - -2.68]**	**0.002**
Gun show	0.06	[-0.63 - 0.74]	0.875	-1.81	[-6.67 - 3.04]	0.464	-1.76	[-6.83 - 3.31]	0.497
May issue	-0.45	[-1.23 - 0.33]	0.259	-0.51	[-4.42 - 3.40]	0.798	-0.96	[-4.60 - 2.68]	0.606
Index of strictness of firearm regulation (deciles of number of total firearm laws)	-0.13	[-0.32 - 0.06]	0.186	0.03	[-0.03 - 0.10]	0.307	-0.1	[-0.25 - 0.06]	0.212

Models were adjusted for the following state-level time-varying variables—population size, proportion aged ≥65 years, race, unemployment rate, poverty rate, the proportion aged ≥25 years without a high school diploma, the rates of crime against property, the percentage of state's population holding a hunting license, licensed gun dealer per 100,000 residents, and the vote share difference between the Republican and Democratic candidates in the presidential election (linear interpolated), in addition to state and year fixed effect.

*: Effect sizes are shown as per 100,000 population.

© 2022 Liu Y et al. *JAMA Network Open*.

eTable 8. Years of Implementation of 3 Selected Laws (2000-2019)

	Laws		
	Universal background checks required at point of purchase for all firearms	All private sellers and licensed dealers are required to keep and retain records of handgun sales	Background checks required for gun show handgun sales at point of purchase
State			
CT	Before 2000	Before 2000	Before 2000
CA	Before 2000	Before 2000	Before 2000
CO	2013	2013	2001
DE	2013	2013	2013
NV	2017	NONE	2017
NY	2013	2013	2000
OR	2015	2015	2000
WA	2014	2014	2014
IL	NONE	Before 2000	2005
VT	2018	NONE	2018
NM	2019	NONE	2019
NJ	NONE	Before 2000	2018

© 2022 Liu Y et al. *JAMA Network Open*.

eTable 9. Number of States That Have Implemented the 9 Laws of Interest

	2000	2010	2019
Background check	3 (6%)	3 (6%)	11 (23%)
Gun show	7 (15%)	9 (19%)	15 (31%)
Permit	4 (8%)	4 (8%)	6 (13%)
Licensed dealer	16 (33%)	15 (31%)	14 (29%)
Records keeping	8 (17%)	8 (17%)	13 (27%)
Straw purchase	2 (4%)	4 (8%)	4 (8%)
May issue	20 (42%)	12 (25%)	8 (17%)
Violence prohibition	1 (2%)	1 (2%)	3 (6%)
Relinquishment	2 (4%)	3 (6%)	6 (13%)

© 2022 Liu Y et al. *JAMA Network Open*.
